# Outcome comparison of rotational ankle fractures: Supination external rotation versus pronation external rotation

**DOI:** 10.1371/journal.pone.0316953

**Published:** 2025-01-16

**Authors:** Min-Su Lee, Gun-Woo Lee, Ji-Hoon Choi, Keun-Bae Lee

**Affiliations:** 1 Department of Orthopedic Surgery, Chonnam National University Hospital, Dong-gu, Gwangju, Republic of Korea; 2 Department of Orthopedic Surgery, Chonnam National University Medical School, Dong-gu, Gwangju, Republic of Korea; University Hospital Zurich, SWITZERLAND

## Abstract

**Background:**

Ankle fractures are among the most common types of fractures in the orthopaedic field, and the Lauge-Hansen classification is commonly used to categorize rotational ankle fractures. This study evaluated and compared the clinical and radiological outcomes of surgically treated supination external rotation (SER) and pronation external rotation (PER) injuries of grades III or IV.

**Methods:**

We retrospectively reviewed and enrolled 104 patients who underwent open reduction and internal fixation for SER or PER injuries classified as Grades III or IV between January 2016 and December 2021, all performed at a single center. Of these, 72 belonged to the SER group and 32 to the PER group. The average postoperative follow-up durations were 31.3 months (range, 24 to 74) for the SER group and 32.1 months (range, 24 to 71) for the PER group. Clinical and radiological outcomes were assessed 24 months after surgery and compared between the two groups. Details of concomitant surgical procedures performed and postoperative complications were also evaluated.

**Results:**

All clinical outcome variables, including the Foot and Ankle Outcome Score, Visual Analog Scale for pain, and ankle range of motion, were comparable between the two groups. Similarly, no statistically significant differences were observed in the development of post-traumatic arthritis or in the frequency of syndesmotic widening 24 months postoperatively. However, the time required for fibular union was significantly longer in the PER group, taking 5.6 ± 2.2 months compared to 3.4 ± 1.3 months in the SER group on average (p < 0.001).

**Conclusions:**

Our study demonstrated that both types of rotational ankle fractures can achieve equivalent clinical and radiological outcomes with surgical treatment. Given the prolonged time to fibular union in the PER group, careful monitoring during postoperative follow-up is required.

## 1. Introduction

Ankle fractures are one of the most common types of fractures in the orthopaedic field, accounting for approximately 10% of all fractures [1–3]. The Danis-Weber classification system classifies ankle fractures based on the location of the fibular fracture line [4, 5]. While it is relatively simple and reproducible, it does not consider the extent of associated ligamentous injuries or damage to medial structures. In contrast, the Lauge-Hansen classification [6, 7] categorizes rotational ankle fractures according to the mechanism of injury. It is divided into four categories: supination external rotation (SER), pronation external rotation (PER), supination adduction (SAD), and pronation abduction (PAB). Subsequent numbers are assigned ranging from grade I to IV, determined by the extent of injury to osseous and ligamentous structures. Higher grades indicate more extensive damage and greater resultant instability.

Among these, the SER injury constitutes the most prevalent type, accounting for approximately 60% of all cases [8, 9]. In advanced stages of SER injury (grade III or IV), fractures or ligamentous injuries extend beyond the distal fibula, involving the posterior malleolus (III) or the medial malleolus (IV). Surgical treatment is frequently required at these stages to address the associated instability[6, 10–12]. Similarly, surgical treatment is necessary in most cases of PER injuries classified as grades III or IV as fractures or ligamentous injuries originating from the medial malleolus progress to involve the distal fibula (III) and posterior malleolus (IV). However, these injuries exhibit different fracture patterns and soft tissue damage compared to SER injuries, due to the difference in foot position at the moment of trauma [8, 13, 14].

Originally designed to aid in the closed reduction of rotational ankle fractures, the Lauge-Hansen classification’s utility has expanded. It is now effectively used for several purposes, including determining the fixation method for fractures and assessing the need for surgical treatment of accompanying syndesmosis or deltoid ligament injuries [15–18]. Numerous studies have addressed the types of fractures and soft tissue injuries that necessitate surgical fixation in rotational ankle injuries, as well as the ideal fixation method [13, 16, 19–24]. However, literature on the differences in outcomes following surgery for each type was scarce [17].

This study aimed to evaluate and compare the clinical and radiological outcomes of surgically treated SER and PER injuries of grade III or IV. We hypothesized that, with surgery performed using appropriate techniques and order of fixation, there would be no significant differences in outcomes between the two groups.

## 2. Materials and methods

This study received approval from our hospital’s Institutional Review Board (CNUH-2023-281) and was conducted in compliance with the World Medical Association’s Declaration of Helsinki guidelines [25]. It also adhered to the Strengthening the Reporting of Observational Studies in Epidemiology (STROBE) guidelines [26]. All data were accessed and collected for research purposes from 01/10/2023 for a duration of three weeks. Informed consent was obtained from all individual participants included in the study through a verbal consent form. We retrospectively reviewed a registry containing 192 patients who underwent open reduction and internal fixation for rotational ankle fractures between January 2016 and December 2021, all performed at a single center. Classification of SER or PER type was primarily determined based on preoperative radiographs and computed tomography (CT) scans and was confirmed through intraoperative findings. Preoperative CT scans were performed for all patients. Of these, injuries classified as SER or PER of grades III or IV met the inclusion criteria. Patients with open fractures, concurrent multiple fractures, a history of previous fractures, or skeletal immaturity (below 16 years of age) were excluded from the study. Patients with a postoperative follow-up period of less than 24 months were also excluded.

**Fig 1:**
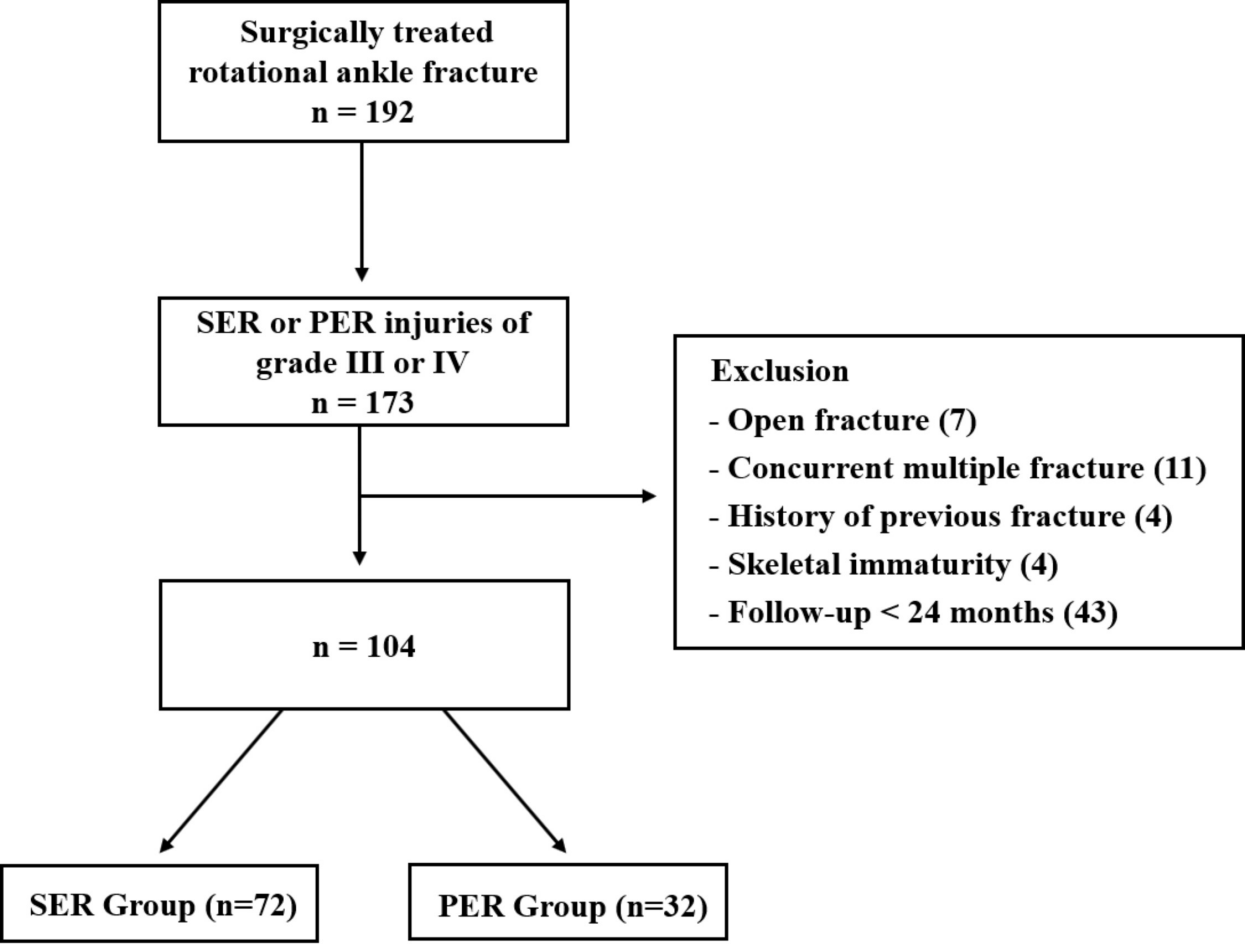
Flowchart of included and excluded patients. SER: Supination external rotation, PER: Pronation external rotation.

Finally, a total of 104 patients met the inclusion criteria and were divided into two groups: the SER group (grade III or IV) and the PER group (grade III or IV) (Fig 1). Data on patient demographics and comorbidities were collected.

### 2.1. Surgical technique and postoperative management

All surgeries were carried out according to injury-specific principles. Upon exposing the fibular fracture site, anatomical reduction was performed. Based on the fracture’s morphology and location, lag screw fixation was performed using a 3.5-mm cortical screw at the surgeon’s discretion. Subsequently, locking plate was used to ensure stable fixation.

After that, fixation of the posterior and medial malleolar fractures was performed as needed. Posterior malleolar fractures were fixed using partially threaded 4.5-mm cannulated screws or small reconstruction plates, while medial malleolar fractures were fixed using the same screws or tension-band wiring [27–29]. After achieving stable bony fixation, syndesmotic stability was assessed through intraoperative stress tests, such as the external rotation stress maneuver or the hook test under fluoroscopy. Syndesmotic instability was diagnosed when the distal tibiofibular overlap measured less than 5mm at 1cm proximal to the tibial plafond or when the lateral displacement of the fibula exceeded 2mm [11, 12, 30, 31]. To treat syndesmotic instability, syndesmotic transfixation was performed using either a 3.5-mm cortical screw or a TightRope (Arthrex, Naples, FL, USA) suture-button device. We routinely assessed medial deltoid ligament injuries using a valgus stress test after achieving anatomic bony fixation and syndesmotic stability. When the widening of the medial clear space was observed on the stress test compared to the opposite side, repair of deltoid ligament was additionally considered.

A short leg splint was applied postoperatively for the first two weeks. Afterward, the splint was changed to a controlled ankle-motion boot to encourage active ankle-motion exercises. Tolerable weight-bearing was allowed at 6 weeks after surgery. All patients were followed up at 1, 3, 6, and 12 months postoperatively and annually thereafter. The syndesmotic screw was removed at least 3 months after the initial surgery. The types of concomitant surgical procedures performed were recorded.

### 2.2. Clinical evaluation

The Foot and Ankle Outcome Score (FAOS) measured 24 months after initial surgery was used as the primary outcome measure, with higher scores indicating superior outcomes and fewer symptoms. The FAOS is a reliable and validated patient-reported outcome measure for foot and ankle disorders that assesses subcategories including symptoms, pain, activities of daily living (ADL), sports, and quality of life (QOL) [32]. The Visual Analog Scale (VAS) for pain, ranging from 0 to 10, was used as a secondary outcome measure, with higher scores indicating more severe pain.

Additionally, the range of motion (ROM) of the ankle and postoperative complications, such as infection, loss of reduction, and nerve injury, were documented. Ankle ROM was recorded by measuring the maximum dorsiflexion and plantarflexion using a goniometer, along the shaft of the fibula and foot. All outcome measures were evaluated 24 months postoperatively, with the clinical evaluation conducted by two independent observers who were not directly involved in the surgical procedure.

### 2.3. Radiological evaluation

Ankle radiographs, including antero-posterior, lateral and mortise views were routinely checked at every follow-up to monitor the status of fracture union and the development of post-traumatic arthritis. Fracture union was defined as the disappearance of the fracture line on follow-up radiographs, without clinical complaints such as pain or tenderness at the fracture site [33]. Cases were categorized as nonunion if union was not confirmed within 9 months after surgery. The degree of post-traumatic arthritis was evaluated 24 months after surgery using the Kellgren-Lawrence (K-L) grade [34].

The presence of syndesmotic widening was also assessed based on radiographs taken 24 months after surgery. Syndesmotic widening was defined as a change exceeding 2mm in the tibiofibular clear space on the antero-posterior view, compared to the initial postoperative weight-bearing radiograph. Two blinded, independent orthopaedic surgeons not involved in the surgical procedure assessed all radiological values using a standard tool in the PACS (Picture Archiving and Communication System: Maroview 6.0; INFINITT Healthcare). In cases of disagreement, the final decision was made by consensus.

### 2.4. Statistical analyses

Data were analyzed using SPSS (version 23.0, IBM Corporation, Armonk, NY, USA). To compare patient demographics, clinical outcomes, and radiological outcomes between the two groups, continuous variables were assessed with an independent t-test, while categorical variables were assessed using Pearson’s χ2 test or Fisher’s exact test. Descriptive statistics were presented as frequencies and percentages for categorical variables and as means ± standard deviations for continuous variables with the percentage and range in parentheses. All statistical analyses were reviewed by a statistician, and p < 0.05 was considered significant.

## 3. Results

Demographic data for the SER and PER injury groups are summarized in Table 1. Among the 104 enrolled patients, 8 were classified as SER III, 64 as SER IV, 15 as PER III, and 17 as PER IV. The SER group comprised 72 patients with a mean age of 49.5 ± 19.8 years, while the PER group included 32 patients with a mean age of 49.1 ± 18.8 years. In the PER group, the proportion of males notably exceeded that in the SER group (52.8% vs 78.1%, p = 0.014). Other variables such as age at the time of injury, body mass index, smoking status, and the presence of diabetes showed no significant differences between the two groups. The average postoperative follow-up durations were 31.3 months (range, 24 to 74 months) for the SER group and 32.1 months (range, 24 to 71 months) for the PER group, respectively.

**Table 1:** Patient demographics.

	SER Group (N = 72)	PER Group (N = 32)	P value
Grade^*^			<0.001
III	8 (11.1)	15 (46.9)	
IV	64 (88.9)	17 (53.1)	
Age^†^ (yr)	49.5 ± 19.8	49.1 ± 18.8	0.917
Gender^*^			0.014
Male	38 (52.8)	25 (78.1)	
Female	34 (47.2)	7 (21.9)	
BMI^†^ (kg/m^2^)	24.7 ± 3.1	24.2 ± 3.2	0.461
Diabetes mellitus^*^	12 (16.7)	3 (9.4)	0.329
Current smoker^*^	4 (5.6)	4 (12.5)	0.220
Follow-up duration^†^ (mo)	31.3 ± 10.4	32.1 ± 9.0	0.873

SER = Supination External Rotation, PER = Pronation External Rotation, BMI = Body Mass Index

^*^Numbers represent the number of ankles, with the percentage in parentheses.

^†^Numbers represent the mean and standard deviation.

### 3.1. Clinical outcomes

The mean FAOS values revealed similar outcomes between the two groups across all subcategories (symptoms, pain, ADL, sports, and QOL). Additionally, no statistical difference was observed in the VAS pain scores between the two groups. Regarding the range of motion in the ankle joint, although the PER group showed slightly inferior results in both dorsiflexion (14.7 ± 6.1 vs 12.9 ± 5.4, p = 0.164) and plantar flexion (34.5 ± 7.3 vs 33.1 ± 6.9, p = 0.342), there was no statistically significant difference (Table 2).

**Table 2:** Comparison of clinical outcomes.

	SER Group (N = 72)	PER Group (N = 32)	P value
FAOS^*^			
Symptoms	79.1 ± 17.6 (53.6 to 100.0)	75.2 ± 14.7 (40.7 to 100.0)	0.329
Pain	80.1 ± 14.7 (41.7 to 100.0)	77.4 ± 14.6 (38.9 to 94.4)	0.575
Activity of Daily Living	88.3 ± 11.0 (60.3 to 100.0)	82.6 ± 13.5 (45.6 to 100.0)	0.565
Sport	69.8 ± 21.8 (35.0 to 100.0)	60.3 ± 17.8 (25.0 to 90.0)	0.277
Quality of Life	63.6 ± 19.6 (37.5 to 100.0)	58.4 ± 15.2 (37.5 to 92.3)	0.332
VAS^*^	2.1 ± 1.2 (0.0 to 5.0)	2.4 ± 1.5 (1.0 to 6.0)	0.740
ROM^*^			
Dorsiflexion	14.7 ± 6.1 (5.0 to 25.0)	13.0 ± 5.0 (5.0 to 20.0)	0.164
Plantarflexion	34.5 ± 7.3 (10.0 to 40.0)	33.1 ± 6.9 (15.0 to 40.0)	0.342

SER = Supination External Rotation, PER = Pronation External Rotation, VAS = Visual Analogue Scale, ROM = Range of Motion

^*^ Numbers represent the mean and standard deviation, with range in parentheses. All outcome measures were evaluated 24 months postoperatively.

### 3.2. Radiological outcomes

**Fig 2:**
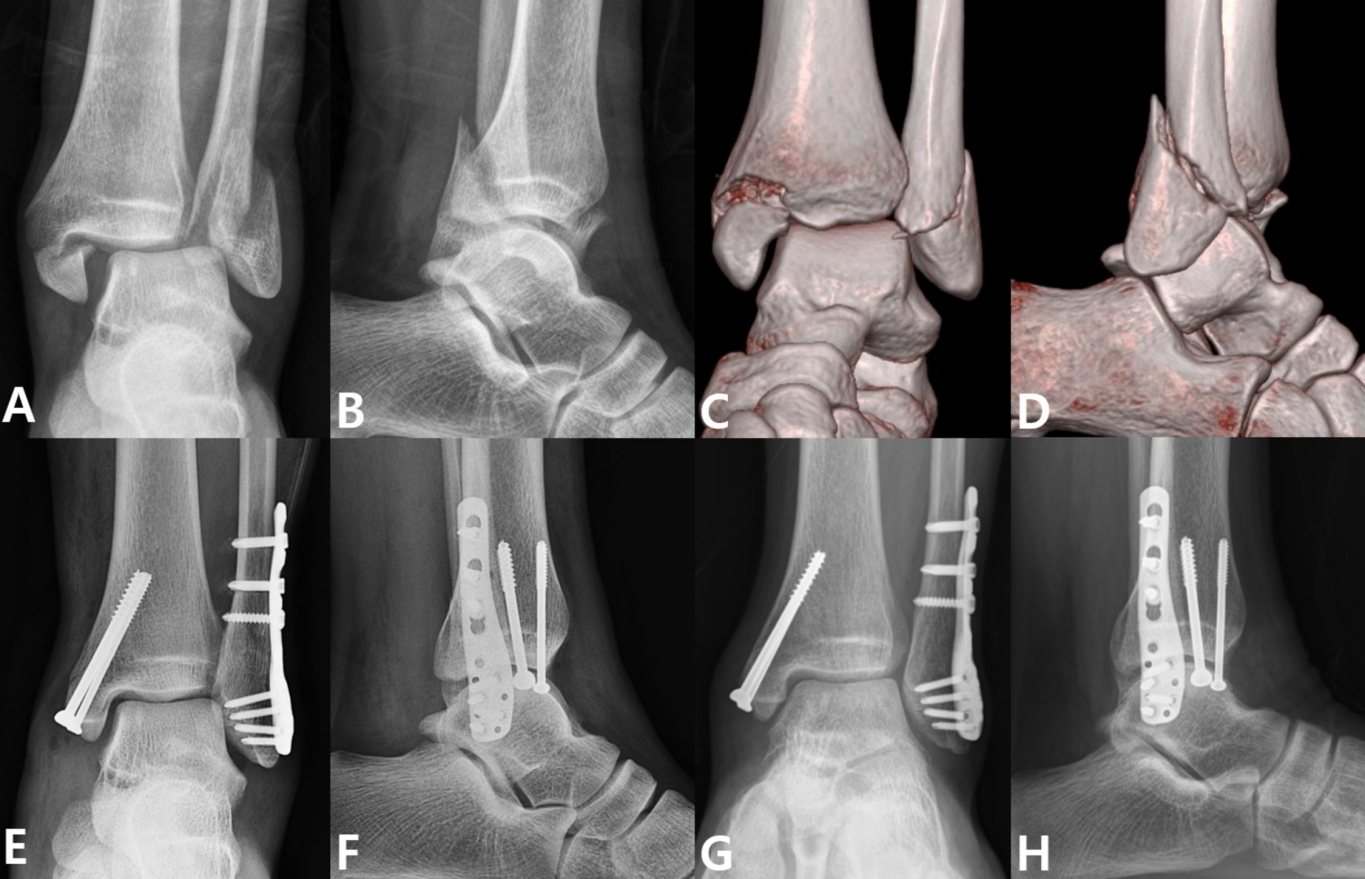
Anteroposterior (A) and lateral (B) radiographs of a supination external rotation ankle fracture. 3D reconstructed anteroposterior (C) and lateral (E) computed tomography images showing an oblique fibular fracture at the syndesmosis level. Anteroposterior (E) and lateral (F) radiographs taken one month after surgery showing anatomical reduction and internal fixation. Anteroposterior (G) and lateral (H) radiographs taken 24 months after surgery showing successful bone union without any perioperative complications.

**Fig 3:**
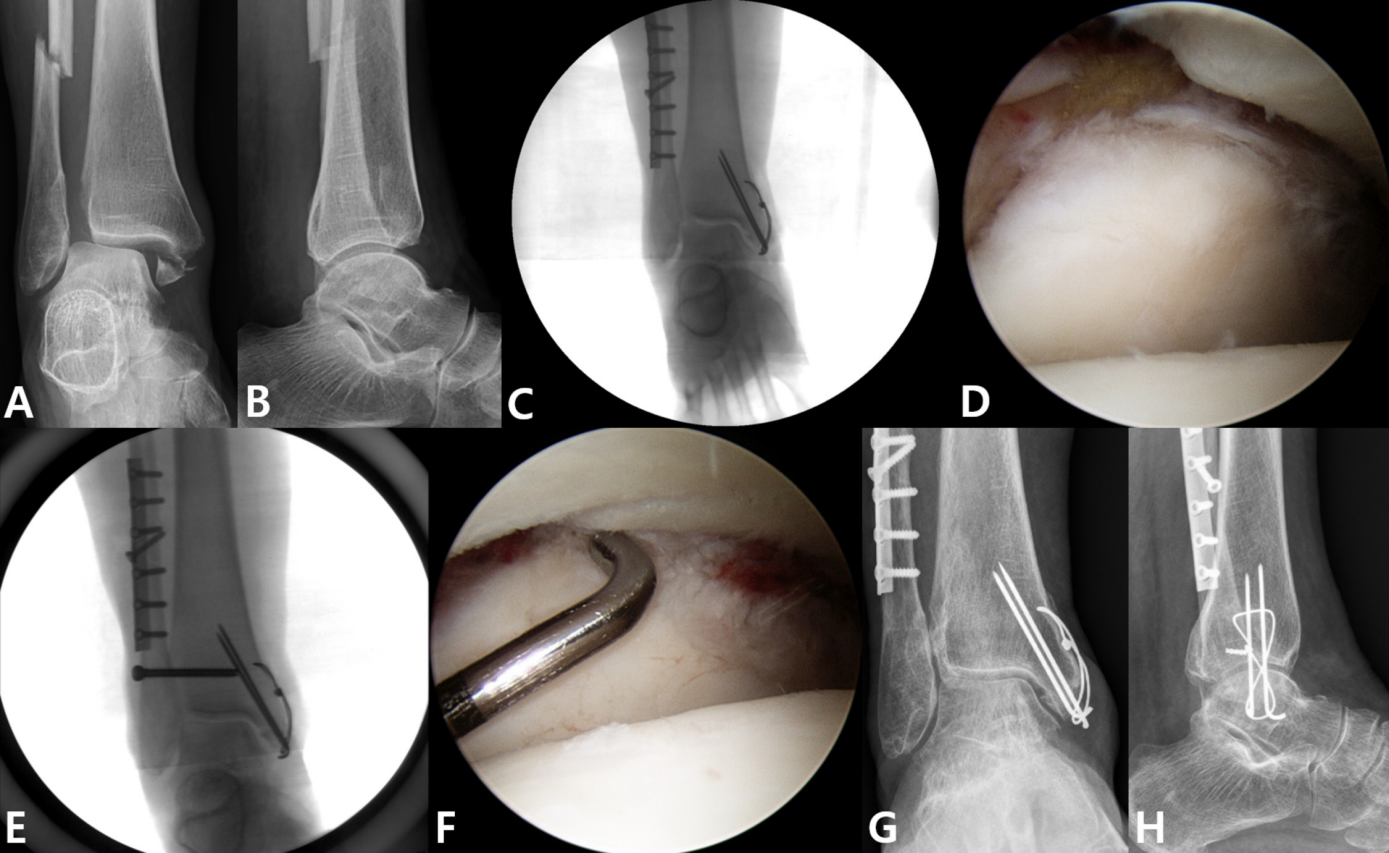
Anteroposterior (A) and lateral (B) radiographs of a pronation external rotation ankle fracture. After confirming anatomical reduction and internal fixation on the intraoperative fluoroscopic image (C), syndesmotic instability was observed through an external rotation stress test performed during arthroscopy (D). After syndesmotic transfixation (E), the stability of syndesmosis was restored (F). The syndesmotic screw was removed after three months, and anteroposterior (G) and lateral (H) radiographs taken 24 months after surgery showing successful bone union without syndesmotic widening.

The K-L grade was used 24 months after surgery to evaluate the development of post-traumatic arthritis, with both groups showing comparable outcomes (Table 3). The proportion of syndesmotic widening cases was relatively higher in the PER group, but this did not reach statistical significance (4.2% vs 12.5%, p = 0.117). Regarding time to bone union, the PER group experienced a significantly longer duration from surgery to fibular union compared to the SER group (3.4 ± 1.3 vs 5.6 ± 2.2 months, p < 0.001). No significant difference was observed between the two groups in terms of tibial union. Figs 2 and 3 illustrate representative cases of surgically treated SER and PER injuries, including follow-up radiographs, respectively.

**Table 3:** Comparison of radiological outcomes.

	SER Group (N = 72)	PER Group (N = 32)	P value
Time to fibular union^*^ (mo)	3.4 ± 1.3	5.6 ± 2.1	<0.001
Syndesmotic widening^†^	3 (4.2)	4 (12.5)	0.117
Kellgren-Laurence Grade^*^	0.4 ± 0.5	0.5 ± 0.7	0.530
0	45	19	
1	24	11	
2	3	1	
3	0	1	
4	0	0	

SER = Supination External Rotation, PER = Pronation External Rotation

^*^Numbers represent the mean and standard deviation.

^†^Numbers represent the number of ankles, with the percentage in parentheses. All outcome measures were evaluated 24 months postoperatively.

### 3.3. Concomitant procedures

When it comes to the concomitant procedures, syndesmotic transfixation was performed at a significantly higher rate in the PER group (15.3% vs 65.6%, p < 0.001) (Table 4). However, the two groups had no significant difference in the frequency of posterior malleolar fixation (p = 0.768) and medial deltoid ligament repair (p = 0.241).

**Table 4:** Concomitant procedures.

	SER Group (N = 72)	PER Group (N = 32)	P value
Posterior malleolar fixation^*^	20 (27.8)	8 (25.0)	0.768
Syndesmotic transfixation^*^	11 (15.3)	21 (65.6)	<0.001
Deltoid ligament repair^*^	3 (4.2)	0 (0)	0.241

SER = Supination External Rotation, PER = Pronation External Rotation

^*^Numbers represent the number of ankles, with the percentage in parentheses.

### 3.4. Complications

Each group had one case of postoperative wound healing complications (Table 5). However, these cases spontaneously healed with meticulous dressing without additional surgical management. In both groups, no instances of postoperative infection, reduction loss, or nerve injury was reported. Two patients from each group experienced postoperative metal irritation, which spontaneously resolved following metal removal. Although the PER group exhibited a higher incidence of nonunion, this difference did not reach statistical significance. Notably, none of the patients required reoperation due to nonunion.

**Table 5:** Postoperative complications.

	SER Group (N = 72)	PER Group (N = 32)	P value
Wound complication^*^	1 (1.4%)	1 (3.1%)	0.552
Postoperative infection	0	0	–
Reduction loss	0	0	–
Nerve injury	0	0	–
Metal irritation^*^	2 (2.8%)	2 (6.3%)	0.395
Nonunion^*^	1 (1.4%)	2 (6.3%)	0.172

SER = Supination External Rotation, PER = Pronation External Rotation

^*^ Numbers represent the number of ankles, with the percentage in parentheses.

## 4. Discussion

The most important finding of this study is that, although the methods of fixation and frequency of required concomitant procedures differ due to the differences in injury mechanisms, equivalent outcomes can be expected in both types of rotational ankle fractures when surgically treated. In our study, the postoperative clinical outcomes of the PER group were equally satisfactory compared to those of the SER group. Both groups demonstrated similar FAOS scores across all subscales and comparable results in secondary outcome measures, such as VAS pain scores and ankle ROM.

To date, several reports have discussed the surgical outcomes of SER injuries [9, 11, 13, 35]. However, reports on the outcomes of surgically treated PER injuries are relatively scarce and conclusions have varied [18, 36]. Donken et al. [36] reported the average 21.6-year follow-up results of 60 patients with grade III or IV PER injuries, and they reported satisfactory (good or excellent) clinical outcomes in 90% of the cases. However, their study included 15% of cases that were treated conservatively. Another study by Schottel et al. [17] compared 22 operatively treated PER IV injuries with 108 SER IV injuries. In their research, the rates of wound complications, nonunion, or loss of reduction showed no significant difference between the two groups. Satisfactory clinical outcomes, including FAOS and ankle ROM, were also observed in both groups. However, a greater proportion of syndesmosis malreduction was noted in the PER group (P = 0.04).

In our study, the PER group exhibited a notably high rate of syndesmotic instability during surgery, aligning with the findings of previous studies [17, 37]. In such cases, syndesmotic transfixation was always performed. Consequently, although the PER group showed a slightly higher frequency of syndesmotic widening at the two-year follow-up, the difference was not statistically significant.

Furthermore, this difference in the incidence of syndesmotic widening did not result in differences in clinical outcomes. Contrary to the widely held belief that anatomic stabilization of the syndesmosis is crucial for improved clinical outcomes [15, 38], several recent studies have reported no significant correlation between the quality of syndesmosis reduction and clinical outcomes [35, 39, 40]. These studies suggest that, due to the various anatomical variations of the syndesmosis, minor differences in anatomic restoration may not significantly affect clinical or radiological patient outcomes. This conclusion aligns with the findings of our study.

Another notable finding of this study is that the PER group demonstrated a significantly longer duration until confirmation of fibular union compared to the SER group (3.4 ± 1.3 vs 5.6 ± 2.2 months, p < 0.001). Despite the absence of significant differences in known risk factors that may delay bone union in ankle fractures, the PER group experienced a more prolonged delay in fibular union, contrary to previously reported findings [17, 41].

The authors speculated about the causes of these results. Compared to SER type injuries, which typically present a spiral or oblique fibular fracture pattern at the syndesmosis level, PER type injuries often exhibit a simpler fibular fracture pattern at the diaphyseal level. This is characterized by a relatively smaller contact area between the fragments at the fracture site. Consequently, achieving bone union typically required a longer duration in the PER group. Nevertheless, the prolonged union period did not result in inferior clinical outcomes or necessitate re-operation to achieve osteosynthesis.

In our study, the two groups demonstrated comparable outcomes. However, the proportion of grade IV injuries was significantly higher in the SER group than in the PER group (88.9% vs. 53.1%, p < 0.01). This difference in the proportion of more advanced-grade injuries may have affected the baseline comparability between the groups. Therefore, future studies should aim to include a larger patient population to enable comparisons within the same grade or between groups with an equal proportion of injury grades.

Several limitations exist in the current investigation. First, due to the retrospective design of this study, certain data were not recorded. A prospective study is necessary to comprehensively analyze the prognosis. Second, this study contains a relatively small number of PER injury cases. Thus, future research, such as multicenter studies, is required to investigate the differences in the frequency of syndesmotic widening and other aspects between the two groups. Finally, although our study did not reveal significant differences in outcomes or complications between the two groups, we cannot rule out the possibility of significant differences emerging over a longer follow-up period.

## 5. Conclusion

In conclusion, the SER and PER groups, which underwent operative treatment for grade III or IV injuries, demonstrated comparable clinical outcomes with no significant differences in complication rates. To achieve such results, it is important to ensure not only the anatomic reduction of the fracture but also the appropriate management of accompanying ligament injuries, based on an accurate assessment of the injury mechanism before surgery. Furthermore, since fibular union tends to take a longer duration in the PER group, careful inspection during postoperative follow-up is needed.

## Supporting information

S1 Data Raw data. Raw data used in this study.
(XLSX)
